# Effect of stress on spatial working memory and EEG signal dynamics in the follicular and luteal phases of the menstrual cycle in young single girls

**DOI:** 10.1002/brb3.3166

**Published:** 2023-07-24

**Authors:** Malihe Eskandari Torbaghan, Ali Moghimi, Hamid Reza Kobravi, Masoud Fereidoni, Imanollah Bigdeli

**Affiliations:** ^1^ Department of Biology, Faculty of Science Ferdowsi University of Mashhad Mashhad Iran; ^2^ Rayan Research Center for Neuroscience & Behavior, Department of Biology, Faculty of Science Ferdowsi University of Mashhad Mashhad Iran; ^3^ Research Center of Biomedical Engineering, Mashhad Branch Islamic Azad University Mashhad Iran; ^4^ Department of Psychology, Faculty of Educational Sciences and Psychology Ferdowsi University of Mashhad Mashhad Iran

**Keywords:** electroencephalogram, estradiol, memory, menstrual cycle, stress

## Abstract

**Aim:**

Women undergo behavioral changes during the menstrual cycle. This study aimed to investigate the effect of estradiol (Es) on stress and effect of stress on spatial working memory (WM) and also to investigate electroencephalogram (EEG) signal's dynamics in the early and late follicular (EF and LF) and luteal (LU) phases of unmarried girls’ menstrual cycle.

**Methods:**

Stress was induced by presentation of a short (3 min) movie clip. Simultaneous with a memory test and stress induction, EEG, serum Es levels, and galvanic skin response (GSR) were assessed.

**Results:**

Serum Es concentrations were decreased in LF, LU, and EF phases. The mean GSR score decreased after stress induction in all three phases, but it increased in the LF and LU phases versus the EF phase. Spatial WM diminished after stress induction in all three phases, but it increased in the LF phase versus the two phases before and after stress induction. Average power spectrum density in all frequency bands increased after stress induction in the frontal and prefrontal channels in the spatial WM test.

**Conclusion:**

The results showed that stress led to spatial WM dysfunction; however, Es improved spatial WM performance in the LF phase versus the other two phases.

## Graphical abstract

This study aimed to investigate the effect of estradiol (Es) on stress and effect of stress on spatial working memory (WM), and also to investigate EEG signal's dynamics in the early and late follicular (FO) (EF and LF) and luteal (LU) phases of unmarried girls' menstrual cycle.

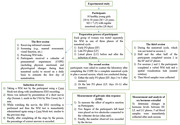

## 1 INTRODUCTION

Women have different physical and mental symptoms during the menstrual cycle (Konishi et al., 2009), they are exposed to more stress and psychological pressures, which may further affect their professional, emotional, and cognitive performances. Stress leads to stimulation of the two system activities that trigger the sympathetic nervous system to secrete catecholamines and initializes the hypothalamic–pituitary–adrenocortical axis activity to release glucocorticoids (GCs). The GCs accompanied by catecholamines control emotional learning and memory processes via affecting receptors that are specially placed in the amygdala and hippocampal structures (de Kloet et al., 2005; Roozendaal et al., 2009). Consequently, stress and stress hormones have significant effects on emotional learning and memory processes and play an important role in the pathogenesis of different mental disorders, such as post‐traumatic stress disorder and anxiety disorders (de Quervain et al., 2017; Merz et al., 2016). So, it is very important to know that the capability to maintain information in memory for a short time is necessary and critical for many cognitive tasks, for example, planning, verbal competence, spatial orientation, mental manipulation of objects, and many others (D'Esposito, 2007; Hyun & Luck, 2007).

Working memory (WM) is a very basic and necessary mechanism for many higher‐order cognitive functions, such as learning and memory, and even mathematical computation (Wilson & Swanson, 2001). The WM structure comprises several components, including the component responsible for the temporary storage of data in modality‐specific buffers and the central executive component that includes a set of tools designed to maintain the active representation of memory tracking, control attention, and protect the latter from irrelevant stimuli‐caused interference (Baddeley, 2003, 2012). A number of neuroimaging studies have shown that the maintenance of data in WM engages a wide network of neural structures, mainly including the prefrontal cortex, and parietal and temporal regions (D'Esposito, 2007; Postle, 2006). Accordingly, several studies have shown that sex hormones can greatly modulate these relationships and networks and help the development, maintenance, and treatment of mental disturbances (Cover et al., 2014; Lebron‐Milad & Milad, 2012; Merz & Wolf, 2017).

For women with a cycle length of 28–29 days, the menstrual cycle may be divided into two general phases, including the follicular (FO) or proliferative phase and the luteal (LU) or secretory phase. The FO phase lasts from day 1 (the first menstruation day) to 14 and is divided into two subphases, including early FO (EF; between the 3rd and 5th day of the menstrual cycle) and late FO phases (LF; between the 12th and 14th day of the menstrual cycle). The LU phase extends from day 14 to 28, or in fact, it is said to be between the 20th and 22nd day of the menstrual cycle (Tsai et al., 1998).

The EF phase is characterized by low estradiol and progesterone serum concentrations. The highest concentration of estradiol is before ovulation, although levels of progesterone remain low; however, the FO phase ends at the ovulation time. The LU phase is characterized by high concentrations of both estrogen and progesterone hormones. Consequently, confirmation of expected hormone concentrations is critical to confirm the menstrual phase, because estradiol fluctuations underlie a reliable pattern of cognitive changes during the menstrual cycle. In other words, this relationship is such that there is a very important relationship among the ovarian secreted estrogen, premenstrual symptoms, menstrual cycle, and ability to perform WM and tasks function (Baddeley & Della Sala, 1996; Konishi et al., 2008). In addition, there are two perspectives on WM performance, including the active retention of information for a certain period of time and the flexible processing of the actively retained information (Osaka & Nishizaki, 2000). Estradiol is one of the factors that may control WM; hence, several studies have reported that memory problems have been observed in postmenopausal women, suggesting that estradiol loss may lead to memory problems. Postmenopausal women have disrupted WM and memory encoding, but not retentive memory, such as storing information for later retrieval (Weber & Mapstone, 2009). Other studies show that both WM and short‐term memory improve in postmenopausal women after estrogen therapy (Duff & Hampson, 2000; Krug et al., 2006; Shaywitz et al., 2003).

Estradiol (E2) and progesterone (P) are two of the most important female sex hormones which have two nuclear receptors α and β (ERα and β) in various regions of the central nervous system, including the hippocampal formation, claustrum, cerebral cortex, amygdala, hypothalamus, subthalamic nucleus, and thalamus (Frick, 2013; Weiser et al., 2008). The secretion of sex hormones is controlled by the hypothalamic–pituitary–gonadal axis which can substantially change in women during the menstrual cycle (Fleischman et al., 2010; Montoya & Bos, 2017) and influence different brain structures, such as the amygdala and the hippocampus via connecting to their corresponding receptors (McEwen & Milner, 2017).

Animal studies using postmenopausal conditions‐mimicking models, such as estrogen replacement‐treated ovariectomized rodents and nonhuman primates, have examined the relationship between cognitive functions and estrogen, providing compelling evidence that ovariectomy and estradiol therapy can affect cognition (Daniel et al., 2006; Lacreuse et al., 2000). In addition, behavioral studies have shown that memory performance may vary even for intact or healthy animals during the menstrual cycle (Lacreuse et al., 2000; Pompili et al., 2010). Several studies have shown that some cognitive abilities, especially WM (Gasbarri et al., 2008; Hampson & Morley, 2013), and visual, verbal, and spatial functions (Hampson et al., 2014; Mordecai et al., 2008; Schöning et al., 2007), can fluctuate during the menstrual cycle due to changes in estrogen levels.

Some studies have shown that during different menstrual cycles and sex hormone levels, the electrophysiological activities of the brain were different in comparison with control groups. Endogenous estradiol regulates the frequency of alpha and theta brain waves in different phases of the menstrual cycle. Analysis of electroencephalogram (EEG) components confirms the effect of estrogen on the functional network of the brain and changes in different phases of the female ovarian cycle. Of many EEG characteristics such as coherence and power of alpha to gamma waves and many brain functional networks, we can name the more important ones that are directly related to cognitive function, such as default mode network, mesoparalimbic network, and sensorimotor network.

Women have poorer memory and more hemispheric asymmetry in the EF phase (low estrogen levels). In this phase, the left hemisphere is more active than the right hemisphere. There is stronger memory and greater hemispheric symmetry in the LF phase (high estrogen levels). Accordingly, E2 and P affect the functional communication of the brain in different phases of the menstrual cycle. E2 increases activity in the left frontal cortex and left parietal lobule, as well as increasing prefrontal connectivity with the hippocampus.

Based on the abovementioned, it can be concluded that one of the most exciting areas of research on women's health is the controlling role of female sex hormones, especially estrogen, in learning and memory function, as well as emotional performance (Andreano et al., 2008; Dolcos et al., 2014; Ferree et al., 2011; Gasbarri et al., 2012; Nielsen et al., 2013; Toffoletto et al., 2014). In order words, extensive studies of cognitive functions have been performed by researchers to identify physiological and pathological changes caused by estrogen (Henry & Sherwin, 2012; Pompili et al., 2012; Poromaa, 2014). It is important to better understand the effect of sex hormone fluctuations on brain structure and functional connectivity that are involved in cognitive, emotional, and self‐referential processing. One of the important and exciting concerns regarding these notes is the attentional and social behaviors of women during their menstrual cycle that may affect marital and/or social relationships. Moreover, these physiological changes and hormonal fluctuations during menstrual cycles in women may act as important effectors when they are in a serious situation for decision‐making.

Therefore, this study aimed to investigate the effect of ovarian cycle estradiol fluctuations on stress and subsequently the effect of stress on spatial WM and also to investigate the dynamic changes of EEG signal in FO and LU phases of the menstrual cycle in young single girls.

## 2 MATERIALS AND METHODS

**FIGURE 1:**
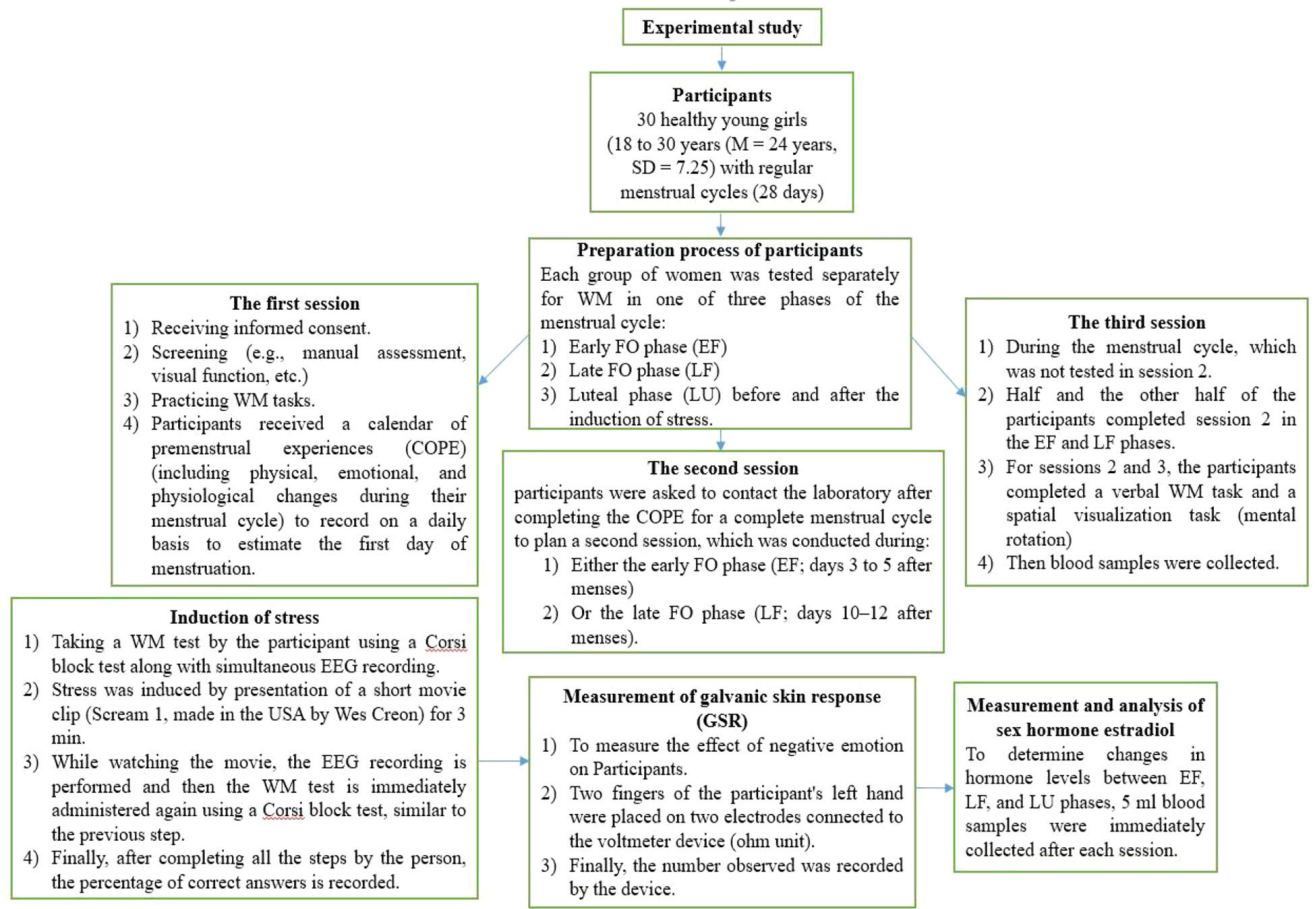
A schematic diagram of the experimental study design.

A summary of the experimental study design in this study is shown in Figure 1.

### 2.1 Ethical approval

The Ethical Review Board of Ferdowsi University of Mashhad, Iran, reviewed and approved the study protocol.

### 2.2 Participants

The statistical population in the study includes 30 healthy young girls. The age range of the participants was 18–30 years (*M* = 24 years, standard deviation [SD] = 7.25), and all participants had regular menstrual cycles (28 days). Inclusion criteria included singleness, right‐handedness, normal vision, and no major medical issues (e.g., neurological or psychiatric diseases). In addition, they had not used contraceptives, corticosteroids, or steroids in the previous 3 months and were not addicted to watching horror movies. After meeting the high criteria, completing the consent form was necessary to participate in the study.

### 2.3 Preparation

Each group of women was tested separately for WM in one of three phases of the menstrual cycle, including the primary or EF phase, the secondary or LF phase, and the LU phase, before and after the induction of stress. Briefly, the preparation process for participants was in three stages: The first session included receiving informed consent as well as screening (e.g., manual assessment, visual function etc.) and practicing WM tasks. During the session, each participant was given a calendar of premenstrual experiences (COPE; Mortola et al., 1990), including physical, emotional, and physiological changes during their menstrual cycle, to record on a daily basis to estimate the first day of menstruation. In the second session, participants were asked to contact the laboratory after completing the COPE for a complete menstrual cycle to plan a second session, which was conducted during either the EF phase (days 3–5 after menses) or the LF phase (days 10–12 after menses, which was confirmed by estradiol and progesterone levels). The third session was conducted during the menstrual cycle, which was not tested in session 2. Half and the other half of the participants completed session 2 in the EF and LF phases. For sessions 2 and 3, the participants completed a verbal WM task and a spatial visualization task (mental rotation), and then blood samples were collected.

### 2.4 Induction of stress

Stress was applied by presentation of a short movie clip. The movie clip (Scream 1, made in the USA by Wes Creon) was used for 3 min to induce negative emotions due to its stressful audio and video content. The procedure involves taking a WM test by the participant using a Corsi block test along with simultaneous EEG recording. The participant is then asked to sit still and watch the movie. While watching the movie, the EEG recording is performed and then the WM test is immediately administered again using a Corsi block test, similar to the previous step. Finally, after completing all the steps by the person, the percentage of correct answers is recorded.

### 2.5 Measurement and analysis of sex hormone estradiol

This method is performed according to the method of Merz et al. (2012) with slight modifications. To determine changes in hormone levels among EF, LF, and LU phases, 5 mL blood samples were immediately collected after each session. The blood sample was poured into blood collection tubes, centrifuged, the serum separated, and stored at −20°C until assayed. Estradiol was evaluated via a solid‐phase, competitive chemiluminescent enzyme immunoassay using an Immulite 1000 (Siemens Healthcare Diagnostics) based on the manufacturer's guidelines or commercially available enzyme‐linked immunosorbent assays for estradiol (Demeditec) to measure free hormone concentrations. Antibody‐coated beads were added to serum and then incubated at 37°C for up to 70 min. Counts per second for each serum sample were converted to analyte concentrations by stored master curves. The assay sensitivity for estradiol was 15 pg/mL. The intra‐ and inter‐assay coefficients of variations were routinely between 5%–8% and 10%–13%, respectively. All data were evaluated by the use of SPSS 20.0 software (SPSS Inc.) with the significance level set to *α* = .05.

### 2.6 Measurement of galvanic skin response (GSR)

The methods of Bradley et al. (2001) to measure galvanic skin response (GSR) and the effect of negative emotion after the application of negative emotion in the study were used. In summary, two fingers of the participant's left hand were placed on two electrodes connected to the voltmeter device (Ω unit) (Electroazin Co.), and finally, the number observed was recorded by the device.

### 2.7 WM test

The Corsi block test is used to measure spatial WM. Briefly, during the WM test, the Corsi blocks appear on the computer screen. Instead of using auditory numbers and names, the location is considered, which consists of nine blue squares, and the candidate should specify the sequence of the illuminated squares. The test becomes more difficult step by step, because in order to prevent repeated search solutions, the lighting of squares and the number of illuminated squares will change randomly. The participants should choose the correct answer with the mouse, and after the person takes the test, the percentage of correct answers is recorded. The total score and the score of each correct answer are finally calculated. The duration of this test is usually 3 min, which may be shorter or longer depending on the candidate's response time. At the same time, EEG brain waves were recorded. The percentages of questions of the WM test answered before and after applying negative emotions in three phases, including EF, LF, and LUE, were recorded (Fischer, 2001).

### 2.8 EEG recording and analysis

At the same time as a memory test and stress induction, EEG recording was performed using a 32‐channel Neuroscan recorder and 5 bands, including delta (1–4 Hz), theta (4–8 Hz), alpha (8–12 Hz), beta (13–30 Hz), and gamma (30–70 Hz). The evaluation of EEG analysis in this study was performed according to the method of Pavlov and Kotchoubey (2017), with a slight modification. In summary, EEG was recorded using 19 electrodes arranged according to the 10–20 system, connected to a Mitsar‐EEG 201 amplifier, and referred to the average earlobe. In addition, two additional electrodes were also used for the horizontal and vertical electrooculogram signals, as one of the bioelectric signals to study the movements of eyes and to design and develop auxiliary or assistive devices. EEG data were collected with sampling frequency at 500 HZ, high pass filter at 0.16 HZ, and low pass filter at 40 Hz. Frequency bands for EEG analysis using individual alpha frequency (IAF) were defined as follows: delta = (IAF from 1 to 4 Hz), theta = (IAF from 2.5 to 6 Hz), alpha = (IAF from −2.5 to +2.5 Hz), beta = (IAF from + 2.5 to 30 Hz), and gamma = (IAF from 30 to 70 Hz). IAF was assessed on EEG recorded for 3 min at rest with eyes closed. The recorded raw EEG segments were analyzed over an interval of 500–6500 ms of the delay period which were accompanied by a filter between 0.5 and 30 Hz and a 50‐Hz notch filter. The segments were also subdivided into 2‐s epochs, and a fast Fourier transformation was utilized in each epoch. Ocular artifacts were corrected using independent component analysis followed by visual inspection of the EEG for residual artifacts. All operations were performed in the EEG laboratory toolbox. For each frequency, spectral power density bands were evaluated by the FieldTrip toolbox and analysis of variance (ANOVA), which included factors group (between‐subject), task, and load (within‐subject). All statistical calculations were performed using the SPSS software package (ver. 19).

### 2.9 Data analysis

The score obtained from the memory test was calculated for each participant and all data were expressed as the mean ± SD. At first, the artifacts were removed from the data obtained from the EEG recording. Then, using the MATLAB software package, the average power spectrum density, mean frequency, med frequency, and coherency of the desired areas were also checked. Finally, statistical analysis was performed by one‐way and two‐way ANOVA followed by the Tukey–Kramer test using SPSS (ver. 16.0) and/or GraphPad Prism (ver. 6.00) for Windows (GraphPad Software). A difference of *p* < .05 was statistically considered a significant difference.

## 3 RESULTS

### 3.1 Evaluation of estradiol concentrations

**FIGURE 2:**
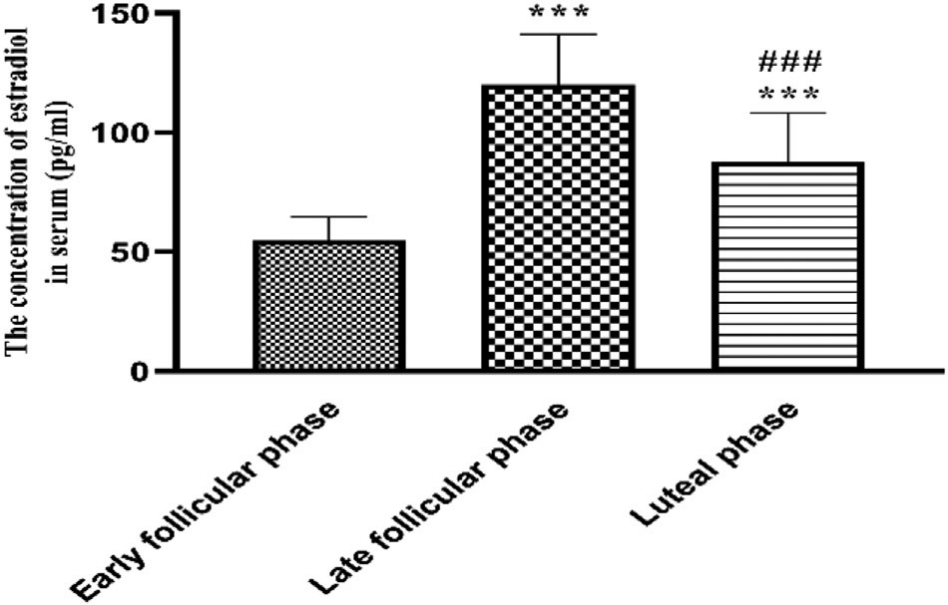
Evaluation of estradiol concentrations during the menstrual cycle in young single girls. Data are shown as mean ± standard deviation (SD), one‐way analysis of variance (ANOVA), and Tukey–Kramer test. ^***^ *p* < .001, compared with the early follicular (FO) and ^###^ *p* < .001, compared with the late FO phase. Early FO (between the 3rd and 5th days of the menstrual cycle), late FO phase (between the 12th and 14th days of the menstrual cycle), and luteal phases (between the 20th and 22nd days of the menstrual cycle). *N* = 30.

As shown in Figure 2, serum estradiol concentrations were considerably higher during the LF phase compared with the EF and LU phases. In addition, serum estradiol levels were greater in the LU phase versus the EF phase, but lower than the LF phase levels (*p* > .001, *F* (2, 87) = 100.4).

### 3.2 Evaluation of GSR score

**FIGURE 3:**
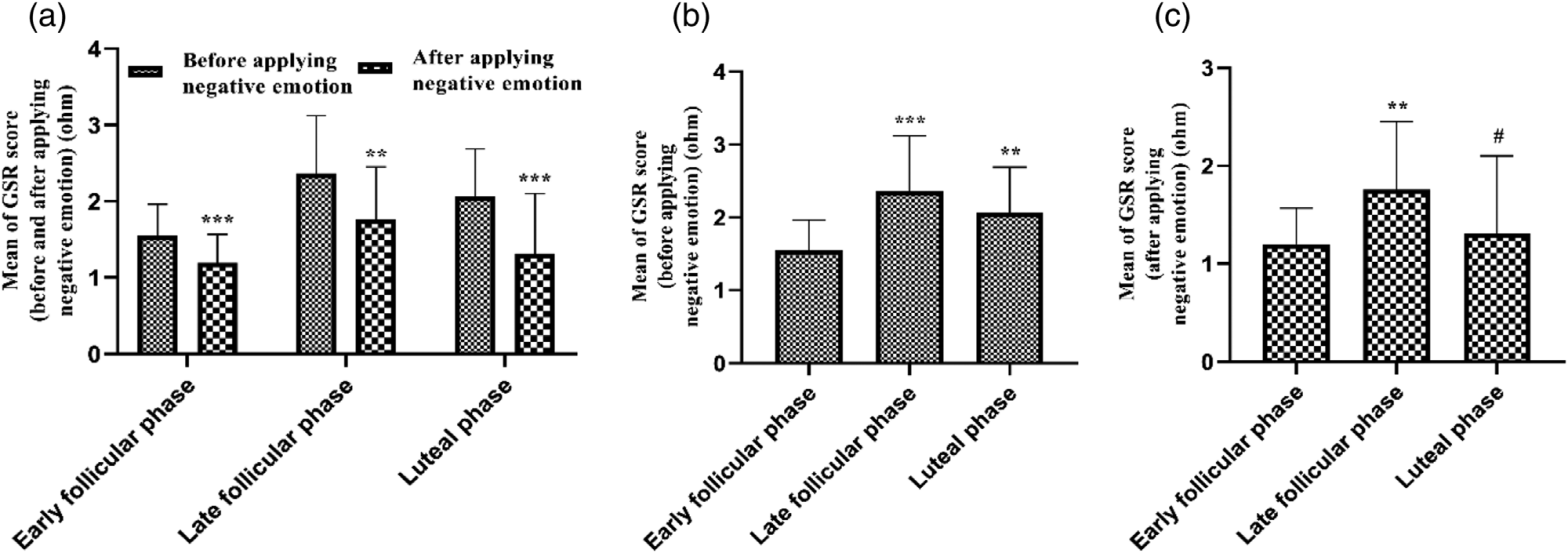
Evaluation of galvanic skin response (GSR) score during the menstrual cycle in young single girls. Data are shown as mean ± standard deviation (SD), one‐way analysis of variance (ANOVA), and Tukey–Kramer test. (a) ^**^ *p* < .01 and ^***^ *p* < .001, compared with before applying negative emotion. (b and c) ^**^ *p* < .01, compared with early follicular (FO) phase; ^#^ *p* < .05, compared with early and late FO phases before and after applying negative emotion. *N* = 30.

In this research, the comparison of the GSR scores recorded from the participants with average emotional intelligence showed that the mean GSR score meaningfully decreased after the negative emotion in all three phases of the menstrual cycle of the participants (*p* > .01 and *p* > .001, *F* (2, 174) = 17.80) (Figure 3a). Moreover, the mean GSR score considerably increased in the LF phase (*p* > .001, *F* (2, 87) = 13.21) and LU phase (*p* > .01) versus the EF phase, but there was no significant difference between the LF phase and LU phase before the negative emotion (Figure 2b). In addition, there was an important difference in the mean GSR scores between the LF phase and EF phase (*p* > .01, *F* (2, 87) = 6.404) and also between the LU phase and LF phase (*p* > .05), whereas there was no significant difference between the LU phase and the EF phase after the negative emotion (Figure 3c).

### 3.3 Evaluation of spatial WM

**FIGURE 4:**
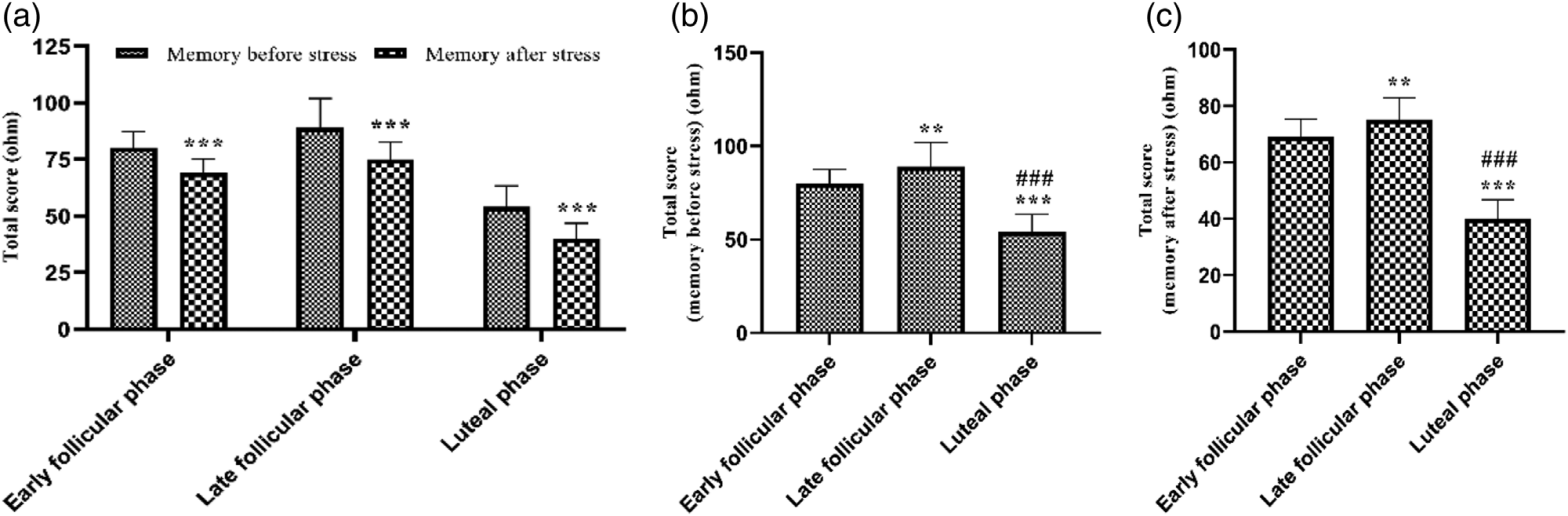
Evaluation of spatial working memory (WM) during the menstrual cycle in young single girls. Data are shown as mean ± standard deviation (SD), one‐way analysis of variance (ANOVA), and Tukey–Kramer test. (a) ^***^ *p* < .001, compared with before applying negative emotion and (b and c) ^**^ *p* < .01 and ^***^ *p* < .001, compared with early follicular (FO) phase; ^###^ *p* < .001, compared with late FO phases before and after applying negative emotion. *N* = 30.

In this research, the results of measuring spatial WM in women with average emotional intelligence, using the Corsi‐block test, indicated that the mean total scores obtained from the spatial WM test significantly diminished after the negative emotional intervention in all three phases of the menstrual cycle of the participants (*p* > .001, *F* (2, 174) = 266.4) (Figure 4a). In addition, the mean total scores considerably increased in the LF phase versus the EF phase (*p* > .01); however, it also reduced in the LU phase compared with early and LF phases before and after the negative emotion (Figure 4b,c) (*p* > .001, *F* (2, 87) = 95.73 and 212.6).

### 3.4 Evaluation of brain dynamics of the EEG spectrum

Regarding EEG changes and characteristics in all three phases (early and LF and LU phases) of the menstrual cycles of the participants, the power spectrum density of different components recorded in the frontal and prefrontal channels showed that the average power spectrum density in all different frequency bands (alpha, beta, delta, gamma, and theta) related to the spatial WM test significantly increased after playing the movie or stress induction (Figure 5a). Nevertheless, no difference was observed between the mean and middle frequencies in any stages of the menstrual cycle (the data are shown in the Appendix). The average frequency after stress induction was not significantly different, which shows that the presence and role of different frequencies (different components) did not affect the signal power and different brain activities. The mean frequency similar to the average frequency did not change significantly either. Therefore, the intervention performed (stress induction using a scary movie) did not change the contribution of frequency components in different rhythms despite the increase in brain processing volume. In addition, as shown in Figure 5b, there are no significant differences between early and LF and LU phases in the average power spectrum density in any frequency bands in the frontal channels; however, there is a significant difference in the average power spectrum density in all frequency bands before and after stress induction in the prefrontal channels.

FIGURE 5(a) Evaluation of average frontal and prefrontal power of the electroencephalogram (EEG) spectrum during the early and late follicular (FO) and luteal phases of the menstrual cycle in young single girls. Data are shown as mean ± standard deviation (SD), two‐way analysis of variance (ANOVA), and Tukey–Kramer test. ^*^ *p* < .05, ^**^ *p* < .01, ^**^ *p* < .01, compared with before playing the movie; ^&^ *p* < .05, ^&&^ *p* < .01, and ^&&&^ *p* < .001, compared with playing the movie; ^###^ *p* < .001, compared with before playing the movie. *N* = 30. (b) Evaluation of average frontal and prefrontal power of the electroencephalogram (EEG) spectrum during the early and late follicular (FO) and luteal phases of the menstrual cycle in young single girls. Data are shown as mean ± standard deviation (SD), two‐way analysis of variance (ANOVA), and Tukey–Kramer test. ^***^ *p* < .001 and ^###^ *p* < .001 compared with early FO phase. *N* = 30.

**Figure:**
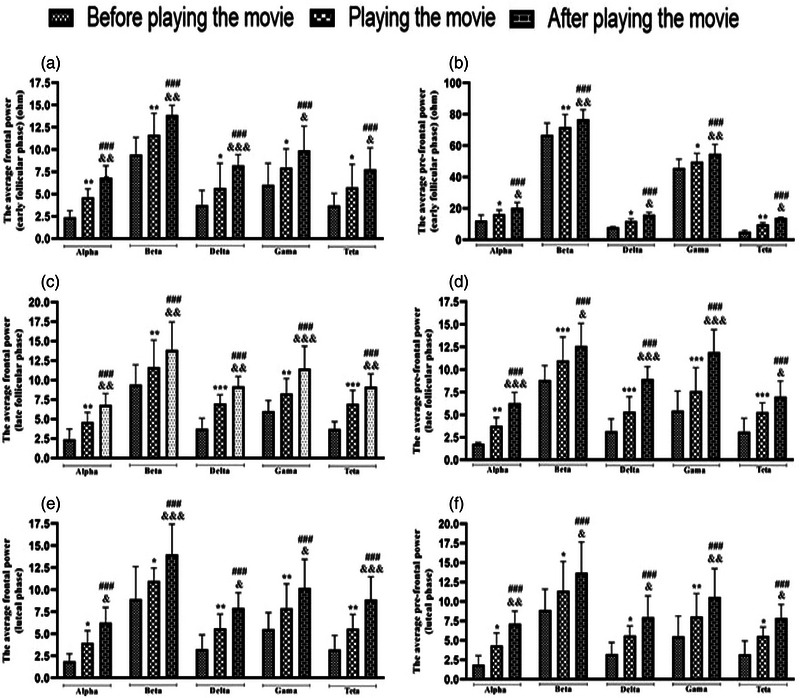

**Figure:**
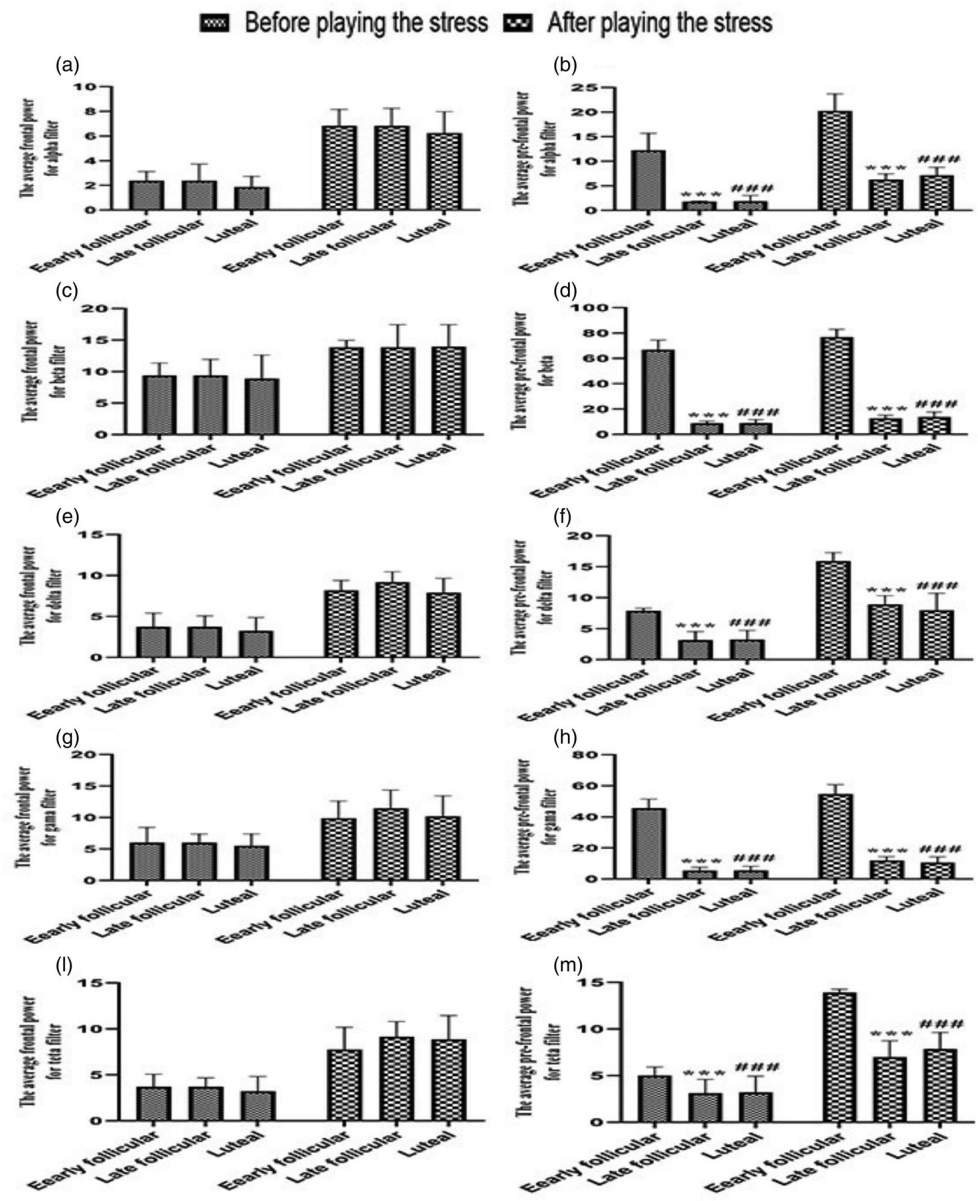

### 3.5 Evaluation of coherency scores

As shown in Table 1, the results of measuring mean coherency in participants with average emotional intelligence displayed that applying different filters, including alpha, beta, delta, gamma, and theta filters, causes no changes in the stages of mean spatial memory coherency scores before and after the negative emotional intervention in any of the three phases of the menstrual cycle of the participants.

**TABLE 1:** Coherency scores (mean and standard deviation [SD]) before and after the negative emotional intervention in all three phases of the menstrual cycle of the participants.

Filter types	Coherency scores before playing the movie (Ω)	Playing the movie (Ω)	Coherency scores after playing the movie (Ω)
	Mean ± SD	Mean ± SD	Mean ± SD
**Alpha**	.99999982 ± .1	.99999997 ± .1	.9999997 ± .2
**Beta**	.999999993 ± .2	.99999992 ± .3	.99999995 ± .3
**Delta**	.9999986 ± .3	.999999965 ± .2	.999999 ± .1
**Gamma**	.99999996 ± .2	.9999996 ± .3	.9999992 ± .1
**Theta**	.9999982 ± .1	.99999996 ± .2	.9999988 ± .3

## 4 DISCUSSION

Studies showed that estrogen fluctuations during the menstrual cycle can affect cognitive and emotional contents and subsequently memory encoding and consolidation processes during emotional interventions. WM is a mental faculty that performs multiple tasks simultaneously and in parallel participates in controlling the distribution of attention with individual differences in attentional capacity. Given that, natural changes in ovarian sex hormones during the menstrual cycle allow researchers to noninvasively investigate estrogen's effects on human cognitive functions (Tsai et al., 1998). Considering this issue, the purpose of this study was to investigate the effect of estradiol surges on stress and subsequently effect of stress on spatial WM, as well as investigating the EEG signal's dynamic variations in the FO (including EF and LF) and LU phases of the menstrual cycle in young single girls.

Accordingly, in this research, the comparison of serum estradiol concentrations recorded from the participants with average emotional intelligence showed that serum E2 concentrations were considerably higher during the LF phase versus the EF and LU phases. Serum E2 levels were also greater in the LU phase versus the EF phase but were less versus the LF phase. Based on the cognitive neuroscience studies the results of numerous concrete examples can be stated in line with our study in this field. Women during the preovulatory phase with high estradiol levels versus the FO phase also showed better spatial learning and memory abilities (Hampson, 1990).

On the one hand, some studies are reporting diverse effects of stress hormones in men and women on a variety of emotional and cognitive processes, for example, in studies involving fear conditioning (Merz et al., 2012, 2013; Stark et al., 2006), reward anticipation (Kinner et al., 2016), or a predictive learning task (Stark et al., 2006). Sex hormones reduce the salivary cortisol levels after stress in men and women (in the FO and LU phases of the menstrual cycle and taking OCs) (Childs et al., 2010; Cornelisse et al., 2011; Espin et al., 2013; Merz & Wolf, 2015; Rohleder et al., 2003). Consequently, stress and sex hormones together participate in modulating emotional and cognitive processes, such as emotional learning, memory processes, and executive functions or motivation (Laman‐Maharg & Trainor, 2017; McEwen et al., 2015; Shields et al., 2016; Shors, 2004). To measure the effect of stress, usually saliva cortisol and GSR are measured. It means that by inducing stress the salivary cortisol levels expressively increase (Childs et al., 2010; Cornelisse et al., 2011; Espin et al., 2013; Merz & Wolf, 2015; Rohleder et al., 2003), while GSR functions meaningfully decrease due to increased sweating (Bradley et al., 2001).

In our study, the GSR method was used to observe the effect of stress on the participants. The results of this study showed that the mean GSR score meaningfully decreased after stress induction in all three phases of the participants’ menstrual cycle. Moreover, although the stress was increased, the mean GSR score considerably increased due to the E2 increase in the LF and LU phases in contrast to the EF phase, therefore, it most likely indicates that probably E2 increased GSR by reducing stress. Increased motivation intensity is usually associated with increased sympathetic activity, so it is plausible that the greatest changes in skin conductance or electrodermal reactions occur when viewing unpleasant or pleasant content compared with neutral ones (Hall & Hall, 2020). Based on this, the results of our study can support that after stress induction, GSR increased due to more E2 in the phases of LF and LU, but less than the LF phase, versus EF phase, leading to better performance of the participants’ WM task. Several studies have found that highly arousing pleasant (erotica) and unpleasant motivational pictures had the largest modulations in emotional response measures, such as physiological arousal (e.g., skin conductance) and defensive reflex activity.

Even so, it has been suggested in numerous studies that temporal proximity is important. It means that stress facilitated memory processes when it is induced within the learning context or shortly before or during encoding induction. However, stress reduced memory performance when it occurred outside the learning context or with a longer time lag between stress and encoding induction, which suggests depending on stress‐induced amygdala activity that modulates hippocampal plasticity (Joëls et al., 2011, 2006; Quaedflieg et al., 2013; Schwabe et al., 2012).

The results of measuring spatial WM in women with average emotional intelligence using the Corsi‐block test indicated that the mean total scores of spatial WM significantly diminished after stress in all three phases of the participant's menstrual cycle. The total mean WM scores also considerably increased in LF versus EF; however, it also reduced in the LU phase compared with EF and LF phases before and after stress induction. According to the results of our study and the results of other studies (Merz & Wolf, 2017), it could be surmised that serum E2 concentrations were meaningfully greater during the LF phase of the women's menstrual cycle when compared with the other two phases (e.g., EF and LU). These results showed the very important role of sex hormones in women regarding the effects of stress on memory as an important cognitive behavior (Merz & Wolf, 2017).

Evidence obtained in the last few decades has shown that brain oscillations in different frequency bands (e.g., alpha, beta, delta, gamma, and theta) provide insight into the processes of attention and memory in humans. Another result of our study was concerning the EEG variations of the participants. In a way, in all three phases of the participants’ menstrual cycles, the average power spectrum density in all different frequency bands related to the spatial WM test significantly increased after stress in the frontal and prefrontal channels. In addition, there is a significant difference among EF, LF, and LU phases in the average power spectrum density in all frequency bands before and after stress in the prefrontal channels.

Among brain frequency bands, two of the most important that were discussed in several studies about attention and memory processes in humans are alpha and beta bands. In neurocognitive research, it has been found that if the amount of information presented to the brain increases, the power decreases (Hanslmayr et al., 2012). The most important effect of this function is related to the alpha band. This means that the increase in the alpha rhythm power and activity during cognitive processing is related to the allocation of attentional resources with the suppression of neural networks, especially cortical areas, unrelated to the current task (Jensen & Mazaheri, 2010; Klimesch et al., 2007; Tuladhar et al., 2007). But it should be stated that this theory is related to alpha oscillations, and its generalization to other brain oscillations could be wrong (Klimesch et al., 2007). Increased beta oscillatory responses are not directly related to mental processes (Deiber et al., 2007; Onton et al., 2005; Siegel et al., 2009), but are directly associated with the amount or quantity of received data (Deiber et al., 2007; Onton et al., 2005; Siegel et al., 2009; Spitzer et al., 2014) and occur when increased brain activity, such as sensory‐specific stimulation of the cortex, increased cognitive load, and viewing of aversive emotional images occurs (Engel & Fries, 2010; Neuper et al., 2009). During auditory stimuli, beta oscillatory responses were increased in central and temporal regions (Haenschel et al., 2000; Mäkinen et al., 2004; Peterson & Thaut, 2002); whereas, during visual stimuli, they increased over occipital electrodes (Senkowski, 2006). Accordingly, beta oscillatory responses play an important role in emotional processes that usually originate from sensory‐specific cortex, current sensorimotor, or cognitive functions, but only when the stimulation is sufficiently effective (Engel & Fries, 2010; Neuper et al., 2009).

Based on the abovementioned reasons, emotional stimulus processing is, however, associated with a reduction or increase of the alpha and beta power bands. In addition, several other studies reported increased alpha/beta power to unpleasant versus neutral or positive pictures (Güntekin & Basar, 2007, 2010; Güntekin & Tülay, 2014; Mennella et al., 2017). It was also observed that the presentation of unpleasant and pleasant pictures increased the amount of interhemispheric beta coherence between the prefrontal and posterior lobes (Güntekin & Başar, 2010; Keil et al., 2007; Miskovic & Schmidt, 2010); however, contrary to this result, it was observed in another study that pleasant images increased beta activity in temporal and parietal areas (Ray & Cole, 1985). These results are in line with our study. The present study displayed that the average power spectrum density in alpha and beta bands, related to the spatial WM test, significantly increased after stress induction in the prefrontal channels, but not in the frontal channels. Differences in results across studies can be partially attributed to different experimental protocols (e.g., duration of stimulus presentation and methodological differences, including evoked than induced oscillations related to an event and different scaling of responses (decibel, percentage, and absolute) and individual differences between different people (e.g., the fundamental lack of attentional resources and loss of motivation) (Başar & Güntekin, 2012; Schubring & Schupp, 2019).

As mentioned earlier, it can be pointed out that the brain is more sensitive to unpleasant, negative, or aversive stimuli, which means that they significantly provoke greater beta (Güntekin & Başar, 2010; Miskovic & Schmidt, 2010; Woodruff et al., 2011) and gamma (Garcia‐Garcia et al., 2010; Keil et al., 2001, 2007; Martini et al., 2012; Oya et al., 2002) power in contrast to pleasant and neutral images. In addition to these EEG bands, numerous studies have found that the theta activity always increased with memory load (Daffner et al., 2011; Itthipuripat et al., 2013; Missonnier et al., 2006; Onton et al., 2005; Pesonen et al., 2007). This sentence can be interpreted as follows: increased frontal midline theta (FMT) power, as the most plausible phenomenon reflecting the activation of the central executive components of WM, during task complexity (e.g., mental manipulations) and executive control demand of memory content, which was in line with abundant studies, indicates positive relationships among FMT, WM process, and cognitive load (Berger et al., 2016, 2014; Cooper et al., 2015; Edin et al., 2009; Ekman et al., 2016; Griesmayr et al., 2010; Palva et al., 2010; Sauseng et al., 2010). It can be speculated that the increased demand for executive control during data manipulation in WM involves a wide network, the main components of which are the prefrontal cortex and ACC (Cashdollar et al., 2009; Eckart et al., 2014; Jeneson & Squire, 2012). EEG variations regarding the gamma and theta bands in our study have displayed that the average power spectral density of the theta band while playing stressful clips versus before and after stress induction significantly increased in all three phases of the participant's menstrual cycle, indicating an increase in WM load.

The results of measuring mean coherency in participants with average emotional intelligence displayed that all rhythms (alpha, beta, delta, theta, and gamma) before and after the stress intervention showed coherency of almost one, indicating that the brain interactions did not change significantly in any of the three phases of the menstrual cycle of the participants. However, the volume of brain processing was high, which emphasizes the improvement of WM. It seems that the non‐change of coherency is due to the range of people who were tested having high intelligence quotient and emotional quotient. Therefore, the amount of brain processing has been high due to the induction of stress, but the brain's ability to simultaneously use visual and auditory information has decreased, as a result, the brain has started a process by increasing its power and activity level to compensate for deficits caused by brain excitement. The results, in other words, confirm increased cortex activity, in which the volume of brain processes has increased to compensate lack of activities in cortical and subcortical tissues. It should be noted that more studies should be conducted in this field.

## 5 CONCLUSION

The goal of the present study was to examine the effect of E2 on stress and subsequently the effects of stress on spatial WM, as well as to investigate the EEG signal's dynamic variations in the FO and LU phases of the young single girls’ menstrual cycles. New findings regarding women with average emotional intelligence revealed that serum E2 concentrations were higher in the LF phase versus the EF and LU phases. Serum E2 levels were also greater in the LU phase versus the EF phase but lower versus the LU phase. The mean GSR score decreased after stress induction in all three phases of the participants’ menstrual cycles, but it increased in the LF and LU phases, especially the LF phase versus the EF phase, suggesting that E2 may modulate stress. Furthermore, spatial WM diminished after stress induction in all three phases of the participants’ menstrual cycles, but it increased in the LF phase versus the two phases before and after stress induction. Average power spectrum density in all different frequency bands (alpha, beta, delta, gamma, and theta) increased after stress in the frontal and prefrontal channels, especially prefrontal channels, related to the spatial WM test. The final results of this study showed that stress led to spatial WM dysfunction; however, E2 improved spatial WM performance in the LF phase versus the other two phases. It should be noted that further studies should be conducted to provide further insight into E2 effects on WM processes and the generalizability of the findings to women at different stages of their menstrual cycle.

## AUTHOR CONTRIBUTIONS

Conceptualization; data curation; formal analysis; investigation; project administration; resources; methodology; software; visualization; writing—original draft; writing—review and editing: **Malihe Eskandari Torbaghan**. Conceptualization; data curation; formal analysis; investigation; methodology; project administration; resources; software; supervision; validation; visualization; writing—original draft; writing—review and editing: **Ali Moghimi**. Conceptualization; data curation; investigation; resources; validation; visualization; writing—review and editing: **Hamid Reza Kobravi**, **Masoud Fereidoni**. Data curation; investigation; methodology; resources; validation; visualization; writing–review and editing: **Imanollah Bigdeli**.

## CONFLICT OF INTEREST STATEMENT

The authors declare no conflicts of interest.

### PEER REVIEW

The peer review history for this article is available at https://publons.com/publon/10.1002/brb3.3166.

## Figures and Tables

**FIGURE A1:**
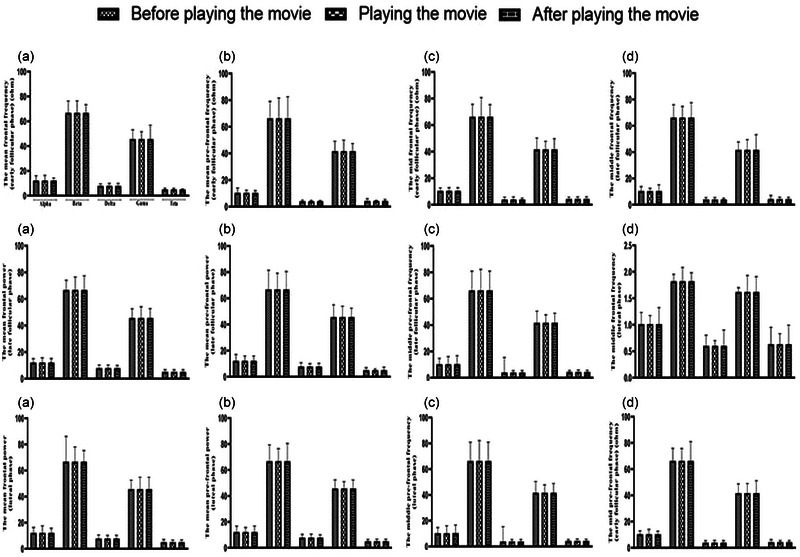
Evaluation of average frontal and prefrontal power of the electroencephalogram (EEG) spectrum during the early and late follicular (FO) and luteal phases of the menstrual cycle in young single girls. Data are shown as mean ± standard deviation (SD), two‐way analysis of variance (ANOVA), and Tukey–Kramer test. *N* = 30.

## Data Availability

The datasets generated during and/or analyzed during the current study are available from the corresponding author on reasonable request.

## 1

Figure A1
